# The regulatory role and therapeutic application of pyroptosis in musculoskeletal diseases

**DOI:** 10.1038/s41420-022-01282-0

**Published:** 2022-12-15

**Authors:** Siyu Wang, Hua Wang, Chengyao Feng, Chenbei Li, Zhihong Li, Jieyu He, Chao Tu

**Affiliations:** 1 Department of Orthopaedics, The Second Xiangya Hospital of Central South University, Changsha, Hunan China; 2 Xiangya School of Medicine, Central South University, Changsha, Hunan China; 3 Hunan Key Laboratory of Tumor Models and Individualized Medicine, The Second Xiangya Hospital of Central South University, Changsha, Hunan China; 4 Department of Geriatrics, The Second Xiangya Hospital of Central South University, Changsha, Hunan China

**Keywords:** Cell death, Diseases

## Abstract

Pyroptosis is a controlled form of inflammatory cell death characterized by inflammasome activation, pore formation, and cell lysis. According to different caspases, pyroptosis can be divided into canonical, non-canonical, and other pathways. The role of pyroptosis in disease development has been paid more attention in recent years. The trigger factors of pyroptosis are often related to oxidative stress and proinflammatory substances, which coincide with the pathological mechanism of some diseases. Pyroptosis directly leads to cell lysis and death, and the release of cytosolic components and proinflammatory cytokines affects cell activity and amplifies the inflammatory response. All the above are involved in a series of basic pathological processes, such as matrix degradation, fibrosis, and angiogenesis. Since these pathological changes are also common in musculoskeletal diseases (MSDs), emerging studies have focused on the correlations between pyroptosis and MSDs in recent years. In this review, we first summarized the molecular mechanism of pyroptosis and extensively discussed the differences and crosstalk between pyroptosis, apoptosis, and necrosis. Next, we elaborated on the role of pyroptosis in some MSDs, including osteoarthritis, rheumatoid arthritis, osteoporosis, gout arthritis, ankylosing spondylitis, intervertebral disc degeneration, and several muscle disorders. The regulation of pyroptosis could offer potential therapeutic targets in MSDs treatment. Herein, the existing drugs and therapeutic strategies that directly or indirectly target pyroptosis pathway components have been discussed in order to shed light on the novel treatment for MSDs.

## Facts

- Pyroptosis is a controlled form of inflammatory cell death.
- The biological characteristics of pyroptosis consist of inflammasome activation, pore formation, and cell lysis.
- The mechanism of pyroptosis can be further classified into canonical, non-canonical, and other pathways based on the presence and subtype of caspases.

## Open questions

- What are the characteristics of different types of cell death?
- What is the interplay between pyroptosis and other forms of cell death?
- What are the underlying mechanisms that pyroptosis is involved in the progression of musculoskeletal diseases?
- Are there any inducers or inhibitors that can target pyroptosis for the treatment of musculoskeletal diseases?

## Introduction

Pyroptosis refers to a programmed cell death featured by caspase-dependent membrane pore formation. Different from non-inflammatory apoptosis, pyroptosis is a form of inflammatory cell death that goes together with the disruption of membrane integrity and proinflammatory intracellular contents release. While this effect is based on regulated membrane pore formation rather than passive membrane rupture, which is the feature of necrosis [1]. Despite the differences in mechanisms, pyroptosis has a complex interaction with other forms of cell death [2]. Since the cause of pyroptosis is often associated with oxidative stress and inflammasome formation, it has been widely discussed in various diseases, such as cancers and neurological diseases, and the associated effects involve cell death and the elicitation of the immune response [3].

Musculoskeletal diseases (MSDs) are a group of locomotor system disorders that affect bones, muscles, joints, and ligaments [4]. MSDs remain a public health burden worldwide, and the risk increases with age [5]. Many inflammatory mediators participate in the occurrence and development of MSDs, including the nucleotide-binding oligomerization domain, leucine-rich repeat (LRR), and pyrin domain-containing 3 (NLRP3), IL-1β, and IL-18, some of which are components of pyroptosis inflammasome or act as upstream or downstream regulators [1]. The effect of pyroptosis on MSDs is mainly mediated by the NLRP3 inflammasome. On the one hand, it accelerates the release of pro-inflammatory cytokines, expands local inflammatory responses, and causes inflammatory damage. On the other hand, through the formation of caspase-1 and Gasdermin(GSDM)D-N, membrane pores can be formed, resulting in cell swelling and dissolution, thus playing a cytotoxic role.

Since several MSDs have been attracting more attention in recent years, we mainly focus on these diseases, including osteoarthritis (OA), rheumatoid arthritis (RA), osteoporosis (OP), gout arthritis (GA), ankylosing spondylitis (AS), intervertebral disc degeneration (IDD), and some muscle disorders. The role of pyroptosis itself and pyroptosis-related proteins in the occurrence and development of MSDs were extensively summarized. Several drugs and treatment strategies have been discussed, although most of them are currently in the experimental stage.

## Mechanisms of pyroptosis

### Molecular mechanism of pyroptosis

The mechanism of pyroptosis consists of the canonical pathway, non-canonical pathway, and other pathways, which are classified as per the presence and subtype of caspases. Canonical pyroptosis is mediated by caspase-1, which is not involved in apoptosis, and the critical event is the activation of canonical inflammasomes. Typically, inflammasomes are comprised of sensors, adapters, and effectors, and they are named after the sensors, which recognize signals directly or through changes in cellular homeostasis [6]. Inflammasomes such as NLRP1/3, NLR family CARD domain containing 4 (NLRC4), and absent in melanoma 2 (AIM2) participate in the canonical pathway [7, 8], among which NLRP3 inflammasome is mostly investigated. NLRP3 inflammasome consists of apoptosis-associated speck-like protein containing a caspase recruitment domain (ASC), NLRP3, and pro-caspase-1, and is activated by two-step signals [9]. The first step is provided by pattern recognition receptors (PRRs) like toll-like receptors (TLRs) and nucleotide-binding oligomerization domain (NOD)-like receptors (NLRs), which recognize the intracellular and extracellular signals, mainly the damage-related molecular patterns (DAMPs) and pathogen-associated molecular patterns (PAMPs), then upregulates the nuclear factor-kB (NF-kB) signals to enhance transcription of proinflammatory components including NLRP3, pro-caspase-1, and cytokines like IL-6/-8/-12, pro-IL-1β/-18. The second step signals include Ca2+, reactive oxygen species (ROS), mitochondrial dysfunction, and lysosomal breakdown [6], which promotes inflammasome assembly and activation. Pro-caspase-1 is transformed into caspase-1 in activated inflammasome, then cleaves GSDMD into GSDMD-C and GSDMD-N. The N-terminal generates membrane pores and leads to cytokine release, K+ efflux, cell swelling, and eventual lysis. Upregulated pro-IL-1β/-18 is conversed to IL-1β/-18 with the help of caspase-1, and thereby released into the extracellular matrix (ECM) through the GSDMD pore. These proinflammatory cytokines also lead to the upregulation of both matrix metalloproteinases (MMPs) and a disintegrin and metalloproteinase with thrombospondin motif (ADAMTS) and finally contribute to ECM destruction [10].

**Fig. 1:**
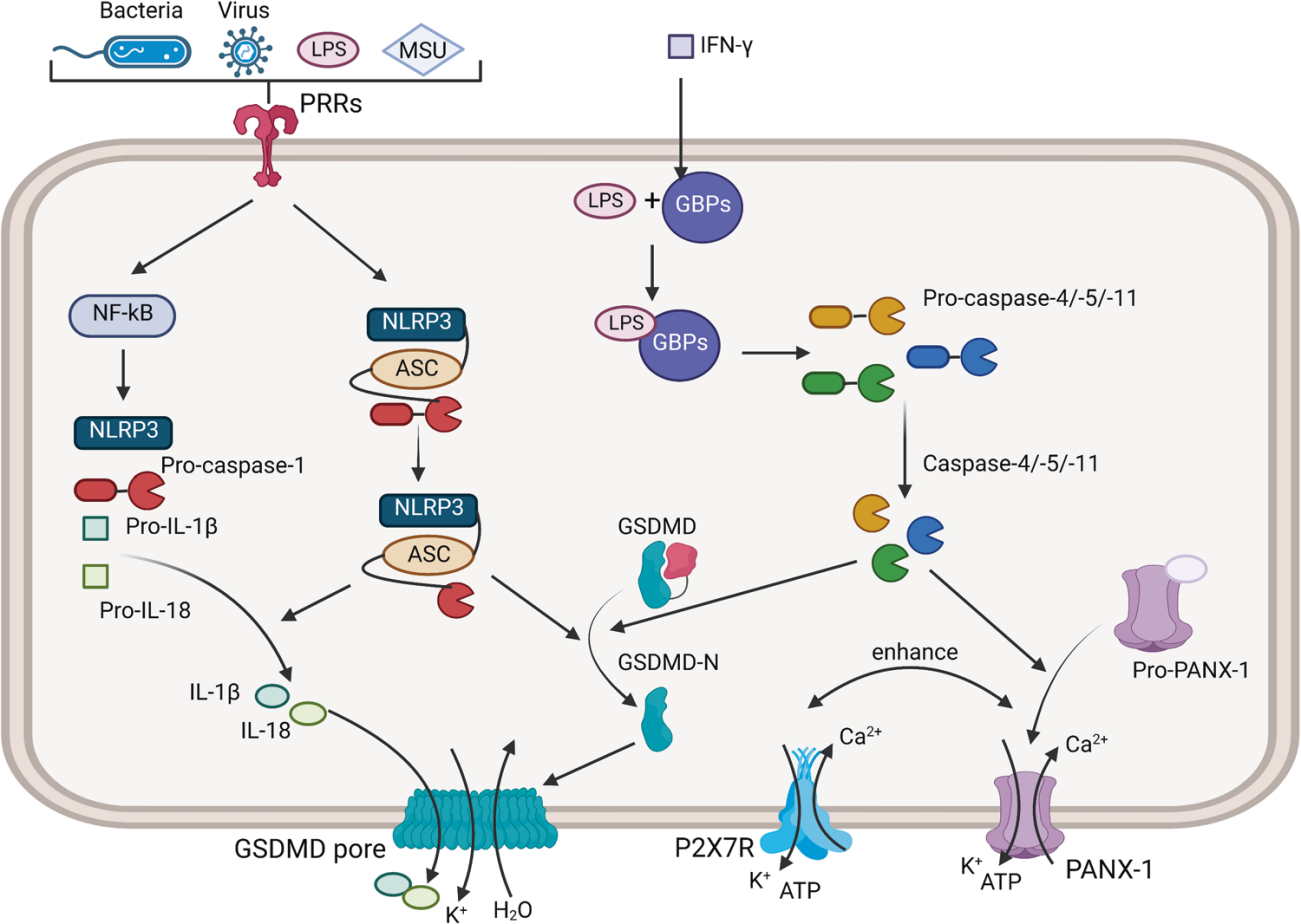
Mechanisms of canonical/non-canonical pyroptosis pathway. Extracellular and intracellular signals like bacteria and virus protein, LPS, and MSU are detected by PRRs and activate NF-kB signaling to upregulate transcription of NLRP3, pro-caspase-1, and pro-IL-1β/-18. Then NLRP3 inflammasome is assembled and activated. Caspase-1 cleaves GSDMD, and the N-terminal generates membrane pores which induce cytotoxicity. Pro-caspase-1β/-18 is also cleaved by caspase-1, and mature cytokines are released through membrane pores. The non-canonical pathway is triggered by intracellular LPS, which activates caspase-4/-5/-11 with the help of GBPs. Activated caspase-4/-5/-11 cleaves GSDMD and pro-pannexin-1 and contributes to membrane pore formation. Pannexin-1 pore has a mutual promoting effect with P2X7 pore, and they lead to K^+^ and ATP efflux and Ca^2+^ influx jointly.

Non-canonical pyroptosis is mediated by the activation of caspase-4/-5 in humans and caspase-11 in mice [8, 11], and these caspases constitute non-canonical inflammasomes alone. While extracellular endotoxin is detected by TLR4 especially, intracellular lipopolysaccharide (LPS) stimulates non-classical inflammasomes directly. Guanylate binding proteins (GBPs), which is induced by interferon (IFN-γ), serve as the responder of cytosolic LPS and provide a platform for the recruitment and activation of caspase-4 [12]. Then GSDMD is cleaved and oligomerized on the membrane, thus leading to a cytotoxic effect. Meanwhile, pro-pannexin-1 is also cleaved to form pannexin-1 (Panx-1) pore, which promotes the outflow of K^+^ and ATP as well as the inflow of Ca^2+^, and further enhances the activation of purinergic P2X7 receptor (P2X7R) and ion channel opening [13]. Yang et al. demonstrated that Panx-1 and P2X7 are downstream of caspase-11, which is required for cytosolic LPS-induced pyroptosis in mice [14], while caspase-4/-5 plays a similar role in humans. It is known that K+ efflux amplifies the activation of NLRP3 inflammasome. Though not cleaving pro-IL-1β/-18 directly, caspase-4/-5/-11 could amplify the inflammatory response and promote caspase-1 function, and the efflux of K+ further promotes NLRP3 activation [15]. To conclude, canonical and non-canonical pathways participate in pyroptosis pathways jointly and share some important regulators [10] (Fig. 1).

**Fig. 2:**
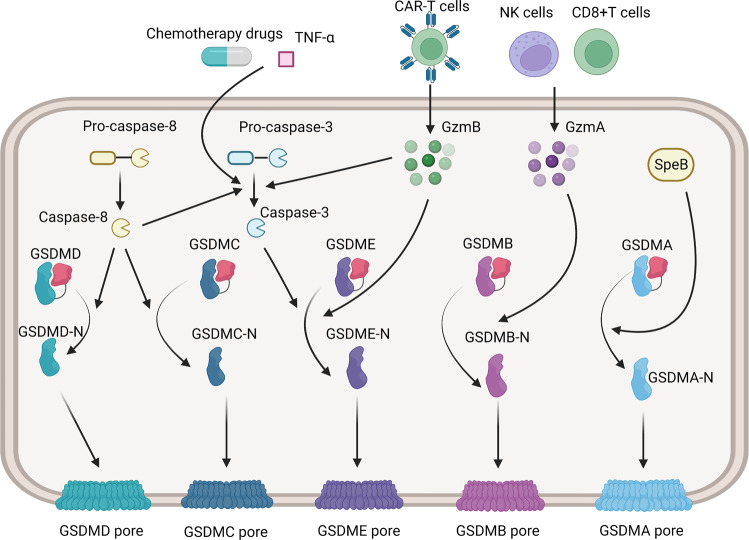
Mechanisms of other pyroptosis pathways. In other pathways, GSDMs are processed by apoptotic caspases, granzymes, and other proteinases. Caspase-3 is activated by certain chemotherapeutic drugs, TNF-α, and caspase-8, then cleaves GADME and forms membrane pores. GSDMD and GSDMC are cleaved by caspase-8 directly. Granzyme B released by CAR-T cells promotes the activation of caspase-3 and cleaves GSDME directly. Granzyme A is released by NK cells and CD8+T cells to cleave GSDMB. GSDMA is cleaved by SpeB in keratinocytes to induce pyroptosis.

Caspase-3/-8, the primary actor of apoptosis, has been confirmed to participate in pyroptosis as well [16, 17]. While GSDMD acts as the critical player in pyroptosis, GSDME, GSDMB, GSDMA, and GSDMC participate in other pathways. Caspase-3 activators include certain chemotherapeutics, TNF-α and caspase-8 [18]. When caspase-3 is activated, the expression level of GSDME determines the mechanism of cell death, and a high level of GSDME switches apoptosis to pyroptosis, which is mediated by GSDME pore [19, 20]. During Yersinia infection, caspase-8 cleaves GSDMD/GSDME and elicits pyroptosis in mice macrophages [17]. Under hypoxia, programmed death-ligand 1 (PD-L1) raises GSDMC expression, and GSDMC is cleaved by caspase-8, thereby transforming TNF-α-mediated apoptosis into pyroptosis in cancer cells [21]. Similarly, metabolite α-ketoglutarate (α-KG) leads to ROS elevation and death receptor 6 (DR6) oxidation, then the latter recruits GSDMC and caspase-8, thus inducing cell pyroptosis [22] (Fig. 2).

Previously, it was believed that the GSDM family was cleaved only by caspases, while two studies in 2020 pointed out that granzyme could also cleave the GSDM family, thus broadening the pathway of pyroptosis. Granzyme B released by chimeric antigen receptor (CAR) T cells directly cleaves GSDME and activates caspase-3 to enhance GSDME pore formation indirectly [23]. Granzyme A, which is released by CD8+ T cells and natural killer (NK) cells, acts in a similar way. The difference is that granzyme B cleaves GSDME while granzyme A cleaves GSDMB, and there is no crosstalk among them [24]. The cleavage of GSDMA is executed by the streptococcal pyrogenic exotoxin B (SpeB) protease of group A streptococcus specifically, and triggers pyroptosis in keratinocytes [25] (Fig. 2). These findings have expanded our understanding of pyroptosis mechanisms.

### Difference and Crosstalk between Pyroptosis and Other Forms of Cell Death

As programmed cell death progress, pyroptosis differs from other cell death pathways morphologically and biochemically. Table 1 provided a concise comparison of the different cell death modes. Apoptosis, the most studied cell death, is characterized by DNA cleavage, chromatin fragmentation, nuclear condensation, cell shrinkage, and membrane blebbing [26]. The wrapped vesicles are then eliminated by phagocytosis. Therefore, apoptosis is considered an active and non-inflammatory process [27]. Necrosis exhibits organelles and cell swelling. As a result of impaired membrane entity, cell content is released, and immune cells are recruited, which is a passive and unregulated process [28]. For a long time, necrosis has been considered uncontrolled cell death, yet studies have shown that necrosis is through complex signal conduction [29]. Necroptosis is defined as a regulated form of necrosis [30]. Both apoptosis and necroptosis can be mediated by death receptor pathways and mitochondrial pathways. Death receptor-mediated pathways begin with the binding of death ligands to the corresponding receptors, then complex I am assembled and triggers the formation of complex II, which induces cell survival, apoptosis, or necrosis depending on the subtype of complex II [31]. Complex IIa activates caspase-8/-10, and the downstream caspases-3/-7 are cleaved and activated, terminating apoptosis [32]. Complex IIb phosphorylates receptor-interacting protein kinase 1 (RIPK1) and RIPK3, then a mixed lineage kinase-like domain (MLKL) is activated and oligomerized, which forms channels on the plasma membrane to mediate necroptosis [33]. The critical event of mitochondria-mediated necrosis and apoptosis is the permeabilization of the inner or outer mitochondrial membrane. Intracellular and extracellular death signals induce the oligomerization of B cell lymphoma-2 (BCL-2)-associated X protein (BAX) and BCL-2 antagonist/killer 1 (BAK), and the oligomers form pores on the outer mitochondrial membrane. Cytochrome c gains access to the cell plasma through the pore and activates caspase-9, then caspase-3/-7 is activated, and mitochondrial apoptosis happens [34]. Elevation of Ca^2+^ concentration in the mitochondrial matrix is the trigger of the mitochondrial permeability transition pore (mPTP) opening on the inner mitochondrial membrane. Opening of mPTP depletes the ion gradient and drives ATP synthesis, resulting in energy deficiency and necroptosis [35]. Another form of proinflammatory programmed cell death, ferroptosis, is characterized by cytosolic iron overload and lipid peroxidation, generally without nuclear pyknosis, and featured smaller mitochondria and increased mitochondrial membrane density [36]. Autophagy is a noninflammatory catabolic pathway that also leads to cell death with intact but amorphous cell membranes. The morphological features include the swelling of organelles and the formation of phagocytic vesicles [37].

**Table 1:** Difference between regulated cell death pathways.

Cell death types	DNA	Nuclear	Cell plasma	Cell membrane	Inflammation	Remark
Apoptosis	Ordered fragmentation	Condensation	Shrink, vesicle wrapped	Intact	×	Apoptosis body generates
Necroptosis	Random fragmentation	Condensation	Swell, release	Rupture	√	–
Pyroptosis	Random fragmentation	Condensation	Swell, release	Rupture	√	Inflammasome activation
Autophagy	Random fragmentation	Condensation	Amorphous, vacuolization	Intact	×	Autophagic vacuoles generates
Ferroptosis	Random fragmentation	Normal	Swell, release	Rupture	√	Shrunken mitochondria, ruptured mitochondrial membrane

Caspase-8 acts as the bridge in the crossing lines of apoptosis, necroptosis, and pyroptosis. The presence of activated caspase-8 favors apoptosis but blocks necroptosis [38]. When RIPK3 is lowly expressed, caspase-8 is automatically activated and initiates the apoptotic pathway [39]. In contrast, when RIPK3 is highly expressed, it interacts with RIPK1 to activate caspase-8; however, the activated caspase-8 further cleaves RIPK1 and RIPK3. Thus, the inactivation of caspase-8 is required for the initiation of necroptosis [40]. As the core event of pyroptosis, the activation of NLRP3 can be attributed to apoptosis and necroptosis. Caspase-8 cleaves GSDMD directly and triggers NLRP3 activation. In death ligand-mediated necroptosis, MLKL-dependent K+ efflux activates NLRP3 [41]. The pore-forming substrates, GSDMD-N and MLKL, induce IL-1β secretion [42]. In A20-deficient macrophages, MLKL facilitates the LPS-induced release of IL-1β, and in vivo experiments indicate that necroptosis plays an important role in inflammasome-dependent arthritis [43]. RIPK1 and RIPK3 have also been identified to activate NLRP3 inflammasome and mediate pyroptosis [44]. The deterioration of RA is associated with the accumulation of synovial macrophages. Lawdor et al. observed that RIPK3 activates NLRP3 and IL-1β inflammatory responses in synovial macrophages of RA model mice, and this effect is independent of MLKL, promoting the chronicity of inflammation and the progression of arthritis [45]. Caspase-3 is another molecular switch that controls apoptosis, pyroptosis, and necroptosis. During apoptosis, GSDME and GSDMD can be directly cleaved by caspase-3, thus inducing pyroptosis or secondary necrosis [46, 47]. Apoptosis of macrophage induces caspase-3 activation and opening of Panx-1 channels, thus activating NLRP3 and promoting pyroptosis [38, 48]. Both pyroptosis and apoptosis of chondrocytes are associated with OA pathogenesis and share an NF-kB signal as the upstream pathway. IL-1β produced by caspase-1 also amplifies inflammation response and apoptosis signals. Yu et al. demonstrated activated NF-kB signal and significantly elevated apoptosis-related proteins, cleaved caspase-1, and pyroptosis markers, cleaved caspase-3, NLRP3, and GSDMD in OA model mice. The above molecules can be downregulated by morroniside with the inhibition of OA progression, while recombinant NF-kB protein reversed this effect, suggesting that apoptosis and pyroptosis of chondrocytes are regulated by the NF-kB pathway [49].

Interestingly, autophagy has been confirmed to regulate inflammatory response and pyroptosis in various disorders. For instance, accumulating evidence showed that nucleus pulposus (NP) cells pyroptosis accelerates IDD progression. Liao et al. demonstrated that moderate autophagy led to the degradation of GSDMD-N and inhibited pyroptosis of NP cells, which was mediated by the autophagy–lysosome [50]. Besides, Wu et al. applied autophagy inducer Betulinic acid to a spinal cord injury mouse model to inhibit pyroptosis and alleviate the injury [51]. Conversely, inhibition of autophagy enhanced hepatocytes pyroptosis induced by As(III) [52]. Besides, ROS-induced autophagy inhibited NLRP3 activation and mitochondria dysfunction in vascular endothelial cells treated with Acrolein [53]. The degree of pyroptosis activation in OA mice is related to autophagy. Yan et al. observed higher levels of pyroptosis markers in OA model mice. The autophagy activator, rapamycin, can reduce the mRNA levels of these markers and alleviate OA at cellular and tissue levels [54]. Autophagy marker light chain 3 plays as the NLRP3 inhibitor to reduce chondrocyte pyroptosis, thus alleviating cartilage degeneration [54]. In addition to pyroptosis, autophagy mainly interacts with apoptosis in MSDs [55]. In the pathogenesis of RA, autophagy antagonizes the apoptosis of fibroblast-like synoviocytes (FLSs) and CD4+ inflammatory T cells, promotes the proliferation of FLSs and chronic self-antigen recognition [56], and further contributes to RA pathogenesis. AKT-related pathways have been targeted to regulate autophagy and apoptosis of RA synovial cells [55]. However, in glucocorticoids-induced cartilage degeneration, autophagy, in response to the ROS/Akt/FOXO3 pathway, reduces ROS in chondrocytes and inhibits their apoptosis, thereby inhibiting the degenerative process of OA and RA [57]. In GA, the crosstalk between autophagy and pyroptosis was mainly mediated by 5’-monophosphate-activated protein kinase (AMPK) and p62. AMPK promotes the autophagy pathway and inhibits the NLRP3 inflammasome in macrophages, while monosodium urate (MSU) inhibits AMPK phosphorylation. Effective mitochondrial autophagy determines the appropriate expression level of p62, thereby inhibiting NLRP3 activation [58]. In conclusion, pyroptosis is not isolated in the process of disease occurrence but is mutually regulated by other death pathways.

## Role of pyroptosis in MSDs

### Pyroptosis and OA

**Fig. 3:**
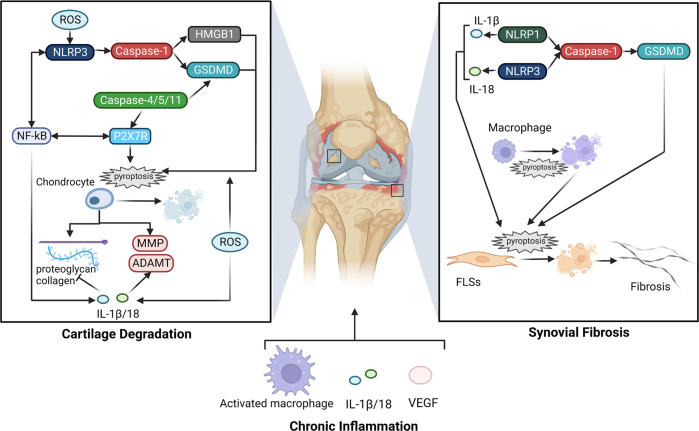
Pyroptosis in OA pathogenesis. The basic pathological processes of OA include cartilage degeneration, synovial fibrosis, and chronic inflammation. On the one hand, excessive ROS can activate NLRP3 inflammasome and downstream caspase-1. On the other hand, it promotes the processing and release of HMGB1, IL-1β/-18, and GSDMD. The activation of caspase-4/-5/-11 and P2X7R in the non-canonical pathway is also involved in this process. NF-kB signal has crosstalk with both canonical and non-canonical pathways, which jointly promote pyroptosis of chondrocytes, imbalance of matrix synthesis and degradation, and release of proinflammatory cytoplasmic components, leading to cartilage degeneration. Synovial fibrosis is mainly mediated by FLSs pyroptosis, in which NLRP1 and NLRP3 participate in the classical pathway and enhance the release of IL-1β/-18, resulting in tissue inflammation. Macrophage pyroptosis also enhances the process of synovial fibrosis. Abnormal angiogenesis is related to cartilage degeneration and synovial inflammation, which is mainly mediated by VEGF. Together with activated immune cells and cytokines, angiogenesis constitutes a chronic inflammatory environment and participates in the occurrence of OA.

OA is one of the most common chronic degenerative diseases worldwide, which is characterized by the proliferation of subchondral bone and age-related degeneration of the articular cartilage [59, 60]. The etiology of OA is complicated with various pathological factors involved, such as occupational/sports activities, joint malalignment, injury, and abnormal loading [61]. Common syndromes, including joint stiffness, pain, and disability, are related to a series of pathological processes, such as synovial fibrosis, ECM destruction, chronic inflammation, and angiogenesis [59]. Recent studies have shown that pyroptosis regulators are involved in OA pathogenesis, implicating novel insights to its treatment strategy (Fig. 3).

Synovial fibrosis, as a fundamental pathological change of OA, is highly correlated with joint stiffness and pain. Its pathogenesis is a combination of inflammation and oxidative stress, in which pyroptosis of fibroblast-like FLSs seems to play a vital role. Zhang et al. reported that NLRPs and related cytokines were upregulated in knee OA synovial and LPS-triggered isolated FLSs. In terms of inducing cytokine activation, NLRP3 and NLRP1 mainly mediated IL-18 and IL-1β activation, respectively [62]. Further research found that increased hypoxia-inducible factor (HIF)-1α aggravated synovial fibrosis in knee OA rats via FLSs pyroptosis, and this effect can be abolished by GSDMD or HIF-1α siRNA [63]. Similarly, another research demonstrated that co-culture with GSDMD siRNA-transfected macrophages could downregulate fibrotic markers in synovial fibroblasts, indicating macrophage pyroptosis was also engaged in the pathological process of knee OA [64].

Cartilage degradation is the consequence of chondrocyte imbalance and ECM destruction. Chondrocytes affect the development of ECM by producing matrix, ECM degrading enzymes, and inflammatory factors like IL-1β, IL-6, and TNF-α [65]. Henceforth, in addition, to affecting their own balance, pyroptosis of OA chondrocytes promotes ECM degradation through disruption of anabolic and catabolic chondrocytes. Non-selective cation channel P2X7 plays a role in non-canonical pyroptosis. Li et al. found that the activation of P2X7R on chondrocytes reduced cell viability and collagen II expressions, while its antagonist downregulated caspase-1, NF-kB, NLRP3, MMP-13, and IL-1β, reduced cartilage degeneration and chondrocyte pyroptosis, and this function is mediated by crosstalk between NF-kB and NLRP3 pathways [66]. Interestingly, the activation state of P2X7R could affect its function. Based on the positive correlation between P2X7 expression and exercise time, Li et al. further found that moderate activation of P2X7R by exercise promoted autophagy and inhibited pyroptosis, thus reducing the OA severity, yet hyper-activated P2X7R may have the opposite effect [67]. Activation of the pyroptosis pathway induces the release of a broad spectrum of cytokines, including IL-1β, TNF-α, and IL-18, which are well known to be associated with cell death and ECM destruction. IL-1β induces the apoptosis of chondrocytes and stimulates the secretion of cartilage-degrading enzymes like MMP-1/-3/-9/-10/-13 and ADMATS-4/-5, which further degrade collagen II and proteoglycans [68, 69]. Moreover, the degraded particles of collagen and proteoglycans could facilitate the production of IL-18 [70]. IL-18 inhibits proteoglycan synthesis and chondrocyte proliferation [71]. Suppressant of NF-kB signaling, like morronside, inhibits chondrocyte pyroptosis and increases collagen II expression, thus attenuating the progression of OA [49].

Excessive ROS leads to cartilage degeneration in terms of reduced chondrocytes and disturbed ECM homeostasis. Directly, oxidative stress to chondrocytes contributes to induced DNA damage and impaired function [72]. ROS is considered a common signal of NLRP3 activation, and mitochondrial dysfunction during pyroptosis also results in the production of ROS [73]. Nitric oxide (NO) acts as an important mediator, which reduces the synthesis of IL-1R antagonists in chondrocytes to increase the susceptibility to other oxidants, enhances the production of MMPs and inflammatory cytokines, and inhibits the synthesis of matrix macromolecules [74]. OA induced by H2O2 is mediated by ubiquitin-specific protease 7 (USP7), which increased the level of ROS, thus aggravating GSDMD-dependent pyroptosis and ECM remodeling [75]. In addition, endogenous ROS also plays a role in OA pyroptosis. Evavold et al. found that ROS triggered by mitochondrial poisons promoted the oligomerization of GSDMD-N and pore formation, though the GSDMD cleavage was independent of ROS function [76]. Stimulated by oxidative stress, transcription factor nuclear factor erythroid-2-related factor 2 (Nrf2) plays a cytoprotective role by regulating the expression of antioxidant proteins. Chen et al. observed upregulated Nrf2, heme oxygenase-1, (HO-1), NLRP3, and ASC levels in both OA synovial samples and the OA rat model. Silence of Nrf2 enhanced ROS and NLRP3 activation [77], suggesting a pivotal role of Nrf2/HO-1 signaling in ROS-induced NLRP3 activation.

OA is accompanied by chronic inflammation, which may aggravate OA pathogenesis and reinforce cell pyroptosis mutually. Upregulated NF-kB and IL-1 family cytokines are the features of the proinflammatory microenvironment. Bauernfeind et al. demonstrated the necessity of NF-kB-dependent signals on NLRP3 expression in mice macrophages, and its expression was confirmed as the limiting factor of its activation [78]. Pyroptosis also amplifies inflammatory reactions by recruiting immune cells and releasing cellular contents, including alarmins, high mobility group protein (HMGB)1, cleaved GSDMD, caspases, and chemokines [79]. Elevated pyroptosis-related inflammatory components were observed in both OA samples and experimental models. A fivefold higher level of NLRP3 protein was found in the synovial membrane of OA patients than that of normal controls [80]. It is accepted that synovial LPS and ATP levels are associated with OA severity and pain [81, 82]. A recent study by Shi et al. isolated FLSs from OA knees after treatment with LPS and ATP, and identified that this effect was mediated by increased levels of NLRP3 inflammasomes and IL-1β/-18 [83]. Uric acid is another stimulator of NLRP3. In a knee OA cohort, Denoble et al. confirmed the positive correlation between the synovial fluid uric acid level and the severity of OA. They also identified IL-1β/-18 produced by NLRP3 inflammasomes as the contributor to OA progression [84]. Downstream products of pyroptosis participate in the inflammatory reaction of knee OA as well. HMGB1 is proven to correlate with cartilage degeneration and synovial fibrosis. Xiao et al. revealed a close association between HMGB1 secretion and FLSs caspase-1-dependent pyroptosis in knee OA rat model, suggesting that HMGB1 production might be attributed to FLSs pyroptosis [85]. GSDMD acts as a critical component in the inflammasome pathway. Yong et al. observed alleviated cartilage degradation, synovial fibrosis, and subchondral bone sclerosis in GSDMD-deficient mice with post-traumatic OA [86]. Antagonists to HMGB1 and GSDMD diminished pyroptosis in LPS-ATP-induced OA chondrocytes, indicating the therapeutic potential by blocking the expression of HMGB1 and GSDMD [87].

Pathological neovascularization occurs in OA cartilage and synovium and disrupts the homeostasis of joints, and the imbalance between proangiogenic and antiangiogenic factors is the major cause [88]. Proangiogenic factors include PG, NO, regulatory peptides, cytokines, chemokines, and growth peptides. Osteochondral or synovial angiogenesis seems to be positively correlated with OA severity, and it may be attributed to activated macrophages in the inflammatory environment [88, 89]. Cuadra et al. treated chondrocytes with IL-1β and observed upregulated VEGF levels, suggesting that IL-1β may promote OA development through the regulation of angiogenesis [90]. Collectively, pathological angiogenesis is often a consequence of chronic inflammation, which affects OA development from the cartilage, subchondral bone, and synovium aspects.

### Pyroptosis and RA

**Fig. 4:**
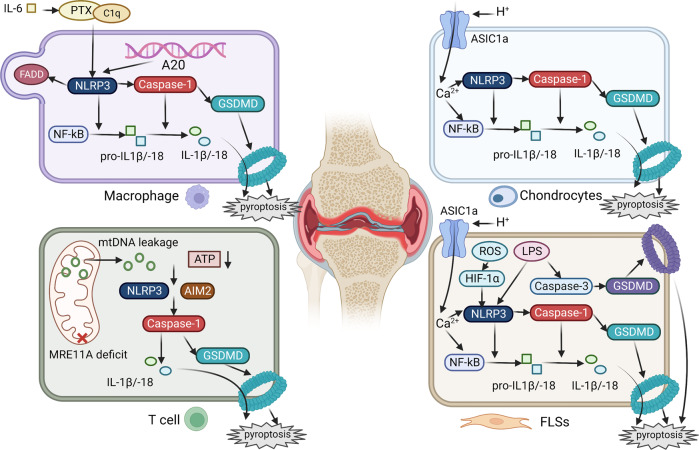
Pyroptosis in RA pathogenesis. RA is characterized by synovitis and cartilage degeneration. Autoimmune components and immune cells are involved in RA. In synovial tissue infiltrating macrophages, NLRP3 is activated by PTX3 and C1q, which is enhanced by IL-6 and regulated by A20. A deficit of mitochondrial gene MRE11A in T cells leads to mtDNA instability and leakage, decreased cytosolic ATP, and activation of NLRP3 and AIM2 inflammasomes, which participate in canonical jointly. Decreased H^+^ in RA synovial fluid promotes the opening of ASIC1a ion channels on chondrocytes and synovial cells, leading to Ca^2+^ influx and activation of NLRP3. In FLSs, the classical pyroptosis pathway is mainly activated by ROS and LPS. LPS also leads to GSDMD cleavage and non-classical pore formation through caspase-3 activation.

RA, a chronic systemic autoimmune disease, is characterized by symmetric and widespread peripheral arthritis, decreased synovial fluid PH, synovial pannus formation, and progressive erosion of the affected joints [91]. Aside from chondrocytes and FLSs, immune cells and related cytokines play a critical role in RA pathogenesis (details concluded in Fig. 4).

Pyroptotic proteins from immune cells are confirmed to be involved in RA pathogenesis. IL-1 is known as a critical mediator in rheumatoid cartilage destruction that is mainly derived from macrophages. To explore the origin of IL-1 in RA, Walle et al. constructed the RA susceptibility gene A20 deficit murine model and observed significant upregulation of NLRP3, caspase-1, and IL-1β in A20-KO macrophages. Furthermore, NLRP3 deletion protected against inflammation and cartilage destruction, suggesting that IL-1β in RA may be produced by NLRP3 inflammasome [92]. CD4+ T cells serve as a proinflammatory effector in RA; Li et al. evaluated the function of DNA repair nuclease MRE11A in RA and found that MRE11A deficiency led to metabolic abnormalities accompanied by ROS, reduced ATP and the leakage of mitochondrial RNA (mtRNA), which further promoted T cell pyroptosis and tissue inflammation [93]. In addition, the fas-associated death domain (FADD) regulates a variety of cellular physiological processes and is identified as an inflammatory biomarker. Mouasni et al. reported FADD secretion as an active process controlled by NLRP3 activation in RA macrophages, which requires K+ efflux, caspase-1, and extracellular glucose. They also found a close correlation between synovial FADD level, inflammatory status, and joint structural damage in RA patients [94]. Moreover, innate immune component serum pentaxin 3 (PTX3) is elevated in RA serum and joint fluid. Wu et al. confirmed the pro-pyroptotic effect of RA serum on monocytes, and also explored the synergy of C1q and PTX3 as well as the driving force of IL-6 in the pyroptotic and inflammatory feedback loop [95].

Activation of the pyroptotic pathway in chondrocytes and FLSs exacerbate RA progression directly. Since increased H+ level in joint fluid is a feature of RA, it is suggested that acid-sensitive ion channel (ASIC)1a may engage in RA pathogenesis by promoting inflammation, synovium proliferation, and osteochondral destruction [96]. Previously, upregulated ASIC1a has been reported to increase Ca2+ influx, activate NLRP3, and in turn, mediate chondrocyte pyroptosis in OA [97]. Recent studies revealed a similar correlation between ASIC1a and RA synovial inflammation as well [98, 99]. In addition to the canonical pathway, studies showed that the caspase-3/GSDMD pathway also mediated pyroptosis of LPS-induced FLSs, and these two pathways were regulated by upstream NF-kB and could jointly induce RA synovitis [100]. Hypoxia is also an important contributor to RA. Hong et al. reported that hypoxia-induced pyroptosis of FLSs through ROS/G protein-coupled receptor kinase (GRK)2/HIF-1α/NLRP3 pathway and HIF-1α inhibitor could block this signal and thus alleviate RA progression [101].

### Pyroptosis and OP

OP is featured by reduced bone mineral density and a higher risk of fragility fractures [102]. The imbalance between osteoclast-mediated bone resorption and osteoblast-mediated bone formation disturbs structural integrity and bone tissue homeostasis [103, 104]. Under stimuli exposure, pyroptosis itself and related inflammatory factors may cause the cellular death or functional inhibition of osteoblasts, and the excessive proliferation and activation of osteoclasts.

Estrogen deficiency and hyperglycemia are considered pivotal triggers in OP progression. In an ovariectomized OP rat model, elevated IL-1, IL-6, and TNF-α were found to inhibit the differentiation of mesenchymal stem cells (MSCs) to osteoblasts [41]. In another similar model, postmenopausal OP rats, inhibition of NLRP3 suppressed inflammatory release and increased osteoblasts number, and bone density, suggesting that NLRP3 may play an important role in OP pathogenesis [105]. Moreover, the diabetes-induced OP rat model showed similar inflammatory factors release and differentiation inhibition, while knockdown or silencing of TLR4 reduced IL-1, IL-6, and TNF-α and subsequently improved osteoblast cell viability [106]. Pyroptosis of osteoblasts may also participate in OP pathogenesis directly. Oxidative stress induced by hypoxia, hyperglycemia, LPS, and ATP stimulation is reported to cause osteoblast pyroptosis and osteogenic dysfunction [107]. Lei et al. reported that pro-inflammatory cytokine IL-17 inhibited proliferation and induced pyroptosis of murine primary osteoblasts in the NLRP3-mediated pathway, which further promoted the release of IL-1β and receptor activator of nuclear factor-kappa B ligand (RANKL) and disrupted bone metabolism [108].

In addition, pyroptosis pathway cytokines also affect osteoclast activity. Osteoclast formation is driven by the appropriate concentration of M-CSF and interaction with RANKL. IL-1β/-18 and TNF-α increase RANKL expression of T cells in a dose-dependent manner, then RANKL triggers osteoclast formation and bone absorption [109]. Among them, TNF-α is considered to be the inhibitor of osteoblasts differentiation and a critical mediator of bone loss [110]. But interestingly, Polzer et al. reported the necessity of IL-1 in TNF-α-mediated inflammatory bone loss since pathological changes were reversed with IL-1 deficiency [111]. In addition, a cross-sectional study also revealed a relationship between IL-1β haplotype and OP susceptibility [112]. IL-18 is also involved in the Th17 cell response to produce IL-17 [113], which plays an important role in OP bone loss. In addition, to directly inducing human monocytes to differentiate into osteoclasts [114], IL-17 can also induce murine dendritic cells (DCs) to transdifferentiate into osteoclast, and this effect can be strengthened by TNF-α and IL-1β [115]. Aside from proinflammatory cytokines production, the osteolysis function of NLRP3 inflammasome can also act in the inflammation-independent pathway, which reorganizes the actin cytoskeleton, and degrades inhibitor of osteoclastogenesis, thus enhancing osteoclast bone resorption capacity [116]. In contrast to the pyroptotic effect of high glucose on osteoblasts, osteoclasts from diabetic mice showed an increased number and bone resorption capacity, which was mediated by the upregulated ROS/mitogen-activated protein kinases (MAPKs)/ nuclear factor kappa-B (NF-κB)/NLRP3 pathway [117]. Similar to NLRP3, NLRC4 also participates in osteoclast differentiation, and bone homeostasis, the absence of each leads to higher bone mass and lower serum IL-1β levels. Unexpectedly, the absence of both did not cause the effect, suggesting a certain interaction between the two targets [118].

### Pyroptosis and GA

GA, an autoimmune disease, is characterized by flares of inflammation, fever, severe pain, and swelling of joints. The cause and detection of gout is the long-lasting hyperuricemia and the deposition of MSU crystals in the synovial fluid. Inflammasomes are evoked by the accumulation of MSU and induced active caspase-1, and IL-1β/-18 [119]. Elevated levels of NLRP3, IL-1β/-18, the risk factors for gout, are detected in serum and synovial fluid of the gouty patients than those of healthy counterparts. The variance in susceptibility to gout is associated with single nucleotide polymorphisms (SNPs) in the NLRP3 inflammasome gene. Only about 10% of patients with hyperuricemia develop GA, demonstrating the importance of NLRP3 in the pathogenesis of GA [120]. There is some crosstalk between NLRP3 activation and the complement cascade. In the MSU-induced complement cascade, C5a release leads to ROS production, which on the one hand, promotes IL-1 production and attracts neutrophils to chemotaxis, and on the other hand, activates the NLRP3 inflammasome and triggers downstream reactions [121].

P2Y14R is a kind of Gi-coupled receptor in macrophages and other immune cells that inhibits cyclic adenosine monophosphate (cAMP) synthesis and is thus considered a potential therapeutic target in GA. The previous study has shown that blockage of the P2Y14R-cAMP pathway suppressed NLRP3-mediated pyroptosis in rat synovial tissue [122]. Similarly, in P2Y14R knockout animal models, cAMP promoted ubiquitination and degradation of NLRP3 to play a key role in cellular pyroptosis. Lack of P2Y14R increased cAMP content, thus improving GA resistance [123]. Another in vivo experiment provided direct evidence of the effect of pyroptosis inhibition in alleviating GA. Tian et al. measured dramatic upregulation of pyroptosis markers, including NLRP3, caspase-1, and GSDMD, in joint tissue homogenate of oxonate- or MSU-induced mice model, while GSDMD antagonist disulfiram suppressed cytokine levels and joint damage via inhibition of pyroptosis [124].

Taken together, the pathogenesis of GA is relatively complex. MSU enhances IL-1β release to promote infiltration of both neutrophils and monocytes [121]. Besides, MSU also induces macrophage necrosis [125]. At the same time, the activation of the complement cascade also participates in the inflammatory response [121], and its interplay with pyroptosis mainly focuses on the NLRP3 inflammasome. All the above evidence reveals that pyroptosis could take part in the crosstalk between MSU-activated gouty and inflammatory cascade.

### Pyroptosis and AS

AS, also termed radiographic axial spondylarthritis, is characterized by structural damage in the sacroiliac joints or spine, vertebral fusion, and bone erosions. Clinical manifestations include reduced mobility of joints and the spine with severe chronic pain. AS has a genetic association with HLA-B27. It is commonly known that its main pathogenesis comprises systemic inflammation and osteogenesis, but the interaction between its genotype and phenotypes still remains to be elucidated [126]. Currently, few studies have explored the relationship between AS and pyroptosis. The details were presented as follows.

Generally, two pathways are located at the end of the immune response: the IL-23/IL-17 axis and the TNF-α axis [126]. By analyzing facet joints specimen of AS patients, IL-23 is found to be secreted by granulocytes, mast cells, and DCs in the subchondral bone marrow and fibrous tissue that replace normal marrow [127]. Under the stimulation of IL-23, IL-17 is produced by Th17 cells. But innate immune cells, mast cells, and granulocytes might be of greater relevance as well [128]. IL-17 production of mucosal-associated invariant cells (MAIT) cells is mediated by IL-7 rather than IL-23 or antigenic stimulation [129]. As to TNF-α, it attributes to microvascular dysfunction and systemic inflammatory reaction [130], and TNF inhibitors could alleviate symptoms and comorbidities [131, 132]. Both two pathways could inhibit bone formation, but the connection and hierarchical order between them remains elusive [127].

It is plausible that pyroptosis pathway participants may affect AS susceptibility. A case–control study confirmed the correlation between SNPs in the IL-1A gene locus and AS susceptibility [133]. Besides, caspase recruitment domain (CARD) 8 acts as an NF-kB inhibitor, and its minor allele is associated with AS susceptibility [134]. In vitro experiments showed that IL-1β/-18 induced the production and secretion of IL-23/-17, which can be blocked by caspase-1 inhibitors [135]. Another case-control study identified the increased NLRP3 inflammasome components in AS synovial fluid [136], and higher levels of IL-23/-17/-1β and NLRP3 were all measured in peripheral blood mononuclear cells (PBMCs) of AS patients [136]. These studies implicate the effect of NLRP3 components and inflammatory cytokines in AS, suggesting that NLRP3 might serve as a potential target of AS.

### Pyroptosis in IDD

The intervertebral disc (IVD) is composed of three components: the cartilaginous endplate (CEP), the annulus fibrosus (AF), and the NP. Unlike articular cartilage, IVD is well-wrapped without vascularization, and the nerves just reach the inner ring [59, 137]. IVD is prone to degeneration due to these characteristics. IDD is caused by cellular and biochemical changes, including pro-inflammatory mediators, progressive ECM loss, altered cell phenotype, and decreased active cells [138].

IL-1β and TNF-α have strong pro-inflammatory activities and are associated with several pathological processes in IDD [139]. IL-1β is secreted by immune cells and IVD cells, which both have a positive correlation with IDD severity degree [140]. In IDD tissue, IL-1β is up-regulated to activate MMPs and ADAMTS to promote ECM degradation. TNF-α may also be involved in stromal catabolism, and it primarily causes disk-derived pain through nerve root stimulation [139]. Of note, IL-1β could promote its expression through the upregulation of NLRP3, and this positive feedback loop is inhibited by melatonin, which shows the potential to treat IDD [141].

Additionally, NLRP3 also has a direct role in IDD. The inflammatory response in this vascular-free tissue might be different from other tissue [142]. In IVD tissues, NLRP3 overactivation leads to increased production of downstream IL-1β and caspase-1, and their expression levels are positively correlated with IDD grade [143]. Recent studies showed that NLRP3 also regulated anabolic and catabolic activity during IDD pathogenesis and up-regulated ECM degradation via MAPK and NF-κB signaling [144]. Oxidative stress may be responsible for NLRP3 activation, and IVD damage since extracellular vehicles (EVs) delivering antioxidant protein could downregulate the pyroptosis of NP cells and retard the progression of IDD [145].

### Pyroptosis and muscle diseases

**Fig. 5:**
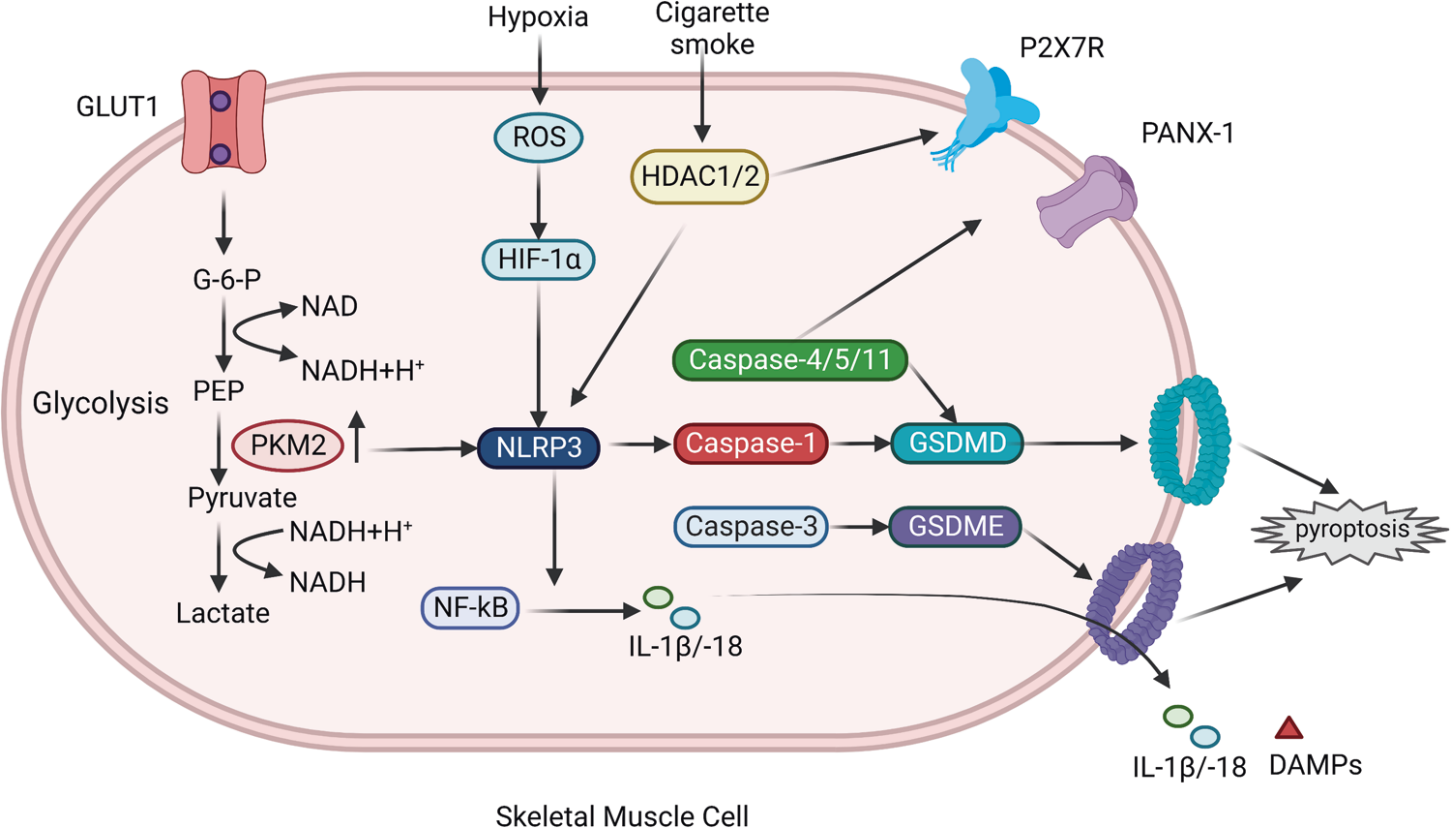
Pyroptosis in Muscle Disorders. Pyroptosis and abnormal function of skeletal muscle cells mediate a series of muscle diseases. The pathogenesis of IIM is associated with glycolysis, and increased PKM2 activity promotes NLRP3 activation. ROS generation induced by hypoxia activates NLRP3 through HIF-1α, and cigarette smoke stimulation enhances HDAC1/2 activity, which is involved in NLRP3, P2X7R and Panx-1 activation. The pyroptosis pathway mediated by caspase-3 and GSDME also leads to membrane pore formation. These pathways interact with NF-KB to promote the release of IL-1β/-18 and DAMPs, amplify tissue inflammation, and thus participate in the pathogenesis.

Myogenesis and regeneration of muscle cause a series of muscle disorders, including idiopathic inflammatory myopathies (IIMs), hypoxia-related muscle injury, dexamethasone (Dexa)- and doxorubicin (Dox)-induced muscle toxicity (DIMT). Previous studies have revealed the positive effect of pyroptosis inhibition on alleviating myocardial and skeletal muscle injury [146, 147]. For IIMs, hypoxia-induced muscle injury, cigarette smoke-induced muscle injury, and drug-induced muscle injury, the potential triggering factors and their effects on myocyte pyroptosis were summarized in Fig. 5.

IIMs, also known as myositis, is a group of systemic autoimmune diseases, mainly including dermatomyositis (DM), polymyositis (PM), and inclusion body myositis. Pathogenesis and clinical manifestations of these subtypes are heterogeneous, but the main and common characteristics are muscle weakness and systemic inflammatory reaction [148]. Previous studies showed that metabolism reprogramming regulates immune responses, especially the glycolysis pathway, and pyruvate kinase (PK) acts as the rate-limiting enzyme [149]. Mass spectrometry analysis showed that PKM2 and other glycolytic proteins are upregulated and co-localized with activated NLRP3 in DM/PM muscle tissues, and the pyroptosis pathway is blocked by PKM2 inhibitor [150]. In mice, experimental autoimmune myositis (EAM) model, expression levels of P2X7R, Panx-1, caspase-4/-5/-11, NLRP3, and GSDMD were higher than that in the control group [13]. A GSDME-mediated mitochondrial pyroptotic pathway leads to perifascicular atrophy in DM muscle, and elevated GSDME is specially cleaved by caspase-3 and converts mitochondrial apoptosis to pyroptosis [151].

Moreover, pyroptosis also mediates other types of skeletal muscle dysfunction. Hypoxia-related muscle injury is crucial to obstructive sleep apnea and its comorbidities. ROS induced by hypoxia could activate NF-kB and HIF-1α pathways and upregulate caspase-1 and GSDMD levels, thus mediating pyroptosis [152]. By increasing histone deacetylase (HDAC) 1/2 level, cigarette smoke exposure promotes skeletal muscle atrophy, morphological changes, and ubiquitin degradation, and regulates pyroptosis biomarkers P2X7R [153]. Similarly, congenital clubfoot muscle phenotype can be induced by FHL1-/y or cigarette smoke exposure during the embryo stage through P2X7R-mediated pyroptosis [154]. The anti-inflammatory agent, Dexa, paradoxically causes muscle inflammation, protein degradation, and muscle atrophy mediated by receptors for advanced glycation end products (RAGE), TLR4, and NLRP3 inflammasome. NLRP3 inhibitors alleviate pyroptosis of skeletal muscle cells and muscle atrophy [155, 156]. DIMT occurs commonly in cardiac muscle and skeletal muscle. In the Dox-treated mice model, TLR4+ cell, NLRP3+ cell, and ASC+ cell increase and take part in the pyroptosis cascade. Reversely, the reduction of these molecules ameliorates clinical manifestation [157, 158]. Therefore, drugs targeting NLRP3 or its upstream molecules can be considered to inhibit pyroptosis and alleviate DIMT [159, 160]. Therefore, studies concerning the inactivation of pyroptotic-related proteins might pave the novel way for the treatment of muscle dysfunction induced by autoimmunity, hypoxia, cigarette smoke, drug toxicity, and other pathological mechanism.

## Potential treatment strategies

At present, a series of drugs and therapeutic strategies targeting MSDs are based on pyroptosis inhibition, including direct blocking of NLRP3 inflammasome and other components of the pyroptosis pathway, and indirect effects on pyroptosis pathway by regulating oxidative stress and inflammatory response. Treatment strategies include blockage of NLRP3, GSDMD, HMGB1, and other components, regulation of endocrine hormones and endogenous cytokines, and application of exosomes and plant extracts, etc. The potential treatments as per the types of diseases were summarized in Tables 2 and 3.

**Table 2:** Common and unique mechanisms related to pyroptosis in MSDs.

Shared pyroptosis pathways	Unique upstream or downstream pathways	Diseases	Biological effects	Target cells	Ref.
NLRP3/Caspase-1/GSDMD, IL-1β, IL-18	LPS-ATP/NLRP3	OA	Fibrosis, synovitis	FLSs	[62]
	HIF-1α/NLRP3				[63]
	NLRP3/Caspase-1/IL-1β, IL-18, HMGB1			Macrophage	[64]
	ATP/P2X7R/NLRP3		Cartilage degeneration	Chondrocytes	[66]
	HA/NLRP3/ MMPs,ADAMTs			Macrophages	[71]
	IL-1, TNF-α/iNOS/NO/NLRP3		Oxidative damage	Chondrocytes	[74]
	H2O2/USP-7/NOX4/ROS/NLRP3				[75]
	LPS/ROS/Nrf2/HO-1/ROS/NLRP3			FLSs	[77]
	Regulator-Rag/mTORC1/mROS/GSDMD		GSDMD oligomerization	Macrophages	[76]
	NF-kB, TLR2,3,4,7/NLRP3		Chronic inflammatory state	Macrophages	[79]
	UA/NLRP3				[84]
	NLRP3/Caspase-1/HMGB1			FLSs	[85]
				Chondrocytes	[87]
	NLRP3/Caspase-1/IL-18	RA	Chronic inflammatory state	Macrophage	[92]
	NLRP3/Caspase-1/FADD				[94]
	PTX3-C1q/NLRP3			Monocyte	[95]
	AIM2/NLRP3			T cells	[93]
	H^+^/ASIC1a/Ca^2+^/NLRP3			Chondrocytes	[97]
	LPS/TLR4/NF-kB/NLRP3			FLSs	[100]
	ROS/GRK2/HIF-1α/NLRP3				[101]
	ROS/MAPKs/NF-kB/NLRP3	OP	Enhanced bone resorption	Osteoclasts	[117]
	LPS/NLRP3				[118]
	MSU/NLRP3	GA	Chronic inflammatory state	Monocytes	[120]
	C5a/ROS/NLRP3				[121]
	P2Y14R/cAMP/NLRP3				[122]
	NLRP3/Caspase-1/IL-1β, IL-18/IL-17,23	AS	Enhanced autoimmunity	T cells	[135]
				Monocytes	[136]
	IL-1β/NF-KB/NLRP3	IDD	Chronic inflammatory state	NPs	[141]
	TNF-α/NAMPT/MAPK, NF-kB/NLRP3				[144]
	PKM2 & NLRP3/Caspase-1/GSDMD	Muscle diseases	Myositis, atrophy	Muscle cells	[150]
	NF-kB/NLRP3, HIF-1α/NLRP3				[152]
	TLR4/NF-kB/NLRP3				[155]
NLRP1/Caspase-1/GSDMD, IL-1β, IL-18	LPS-ATP/NLRP1	OA	Fibrosis, synovitis	FLSs	[62]
	NLRP1/Caspase-1/HMGB1		Chronic inflammatory state		[85]
	NLRP1/Caspase-1/HMGB1		Chronic inflammatory state	Chondrocytes	[87]
NLRC4/Caspase-1/GSDMD, IL-1β, IL-18	LPS/NLRC4	OP	Enhanced bone resorption	Osteoclasts	[118]
Caspase-4,5,11/P2X7R-panx-1	P2X7/AMPK/mTOR	OA	Autophagy ↑ , pyroptosis ↓	Chondrocytes	[67]
	HDAC1,2/P2X7R	Muscle diseases	Myositis, atrophy	Muscle cells	[153]
NF-kB/IL-1β, IL-18	NF-kB/MMP13,IL-1β, TNF-α	OA	Cartilage degeneration	Chondrocytes	[66]
	CARD8/NF-kB	AS	Chronic inflammatory state	-	[134]
	NF-kB/IL-1β, TNF-α/MMPs, ADAMTs	IDD	Chronic inflammatory state	NPs	[140]
Caspase-3/GSDME	LPS/TLR4/NF-kB/Caspase-3/GSDME	RA	Chronic inflammatory state	FLSs	[100]
	Caspase-3/GSDME	Muscle diseases	Myositis, atrophy	Muscle cells	[151]

**Table 3:** Drugs targeting pyroptosis in MSDs.

For Diseases	Drugs	Targets	Mechanisms	Target cells	Ref.
OA	CY-09	NLRP3	Maintain ECM homeostasis and regulate inflammation	Chondrocytes	[165]
	Quercetin		Block oxidative stress-induced chondrocyte pyroptosis		[174]
	Lico A		Inhibit NLRP3 inflammasome, upregulate aggrecan and collagen-II to alleviate ECM degradation		[172]
	Estradiol		Inhibit NLRP3 activation	FLSs	[83]
	SDF-1		Inhibit NLRP3 activation and AMPK pathway		[183]
	Moderate-intensity exercise	P2X7R, NLRP3	Promote autophagy and degrade NLRP3	Chondrocytes	[67]
	melatonin	NF-kB, NLRP3	Antioxidant, scavenge free radical and inhibit inflammation		[97]
	BMSC-Exos/miR-326	NLRP3, NF-kB	Upregulate chondrocyte-specific genes and downregulate pyroptosis related proteins		[168]
	Morroniside		Maintain ECM homeostasis and regulate inflammation		[49]
	disulfiram	GSDMD	Antagonize GSDMD and downregulate pyroptosis		[87]
	glycyrrhizic acid	HMGB1	Antagonize HMGB1 and downregulate pyroptosis		[87]
OP	Insulin	ROS/MAPKs/NF-κB/NLRP3	Decrease glucose level and enhance efferocytosis	Osteoclasts	[117]
	Irisin	Nrf2, NLRP3	Upregulate Nrf2 and inhibit NLRP3 inflammasome	Osteoblasts	[105]
	Simvastatin	BMP-2	Promote the differentiation of MSCs into osteoblasts via BMP-2/Smads signaling		[181]
RA	PUN	NF-kB, NLRP3	Inhibit M1 polarization and pyroptosis	Macrophages	[176]
	MDP	GRK2	Regulate hypoxia induced ROS/GRK2/HIF-1α/NLRP3 pathway	FLSs	[101]
	Amiloride, PcTx-1	ASIC1a	Alleviate calcium influx	Chondrocytes	[97]
	BAPTA-AM	Ca^2+^	Chelate calcium		[97]
	VX-765	Caspase-1	Pharmacological blockade of caspase-1 activity	T cells	[93]
GA	Forskolin	P2Y14R	Activate adenylate cyclase and increase cAMP level	Macrophages	[123]
IDD	Melatonin	NLRP3	Inhibit NF-κB signaling and downregulate mtROS production	NPs	[141]
	dECM@exo		Regulate MMPs and inflammatory reaction		[171]
	Exosome/ miR-26a-5p		Degrade METTL14 and inhibit NLRP3 activation		[170]
	Cavin-2-modified engineering EVs	ROS	Provide antioxidant protein		[145]
Muscle diseases	Glyburide and BBG	NLRP3, P2X7R	Reduce the expression levels of NLRP3 and P2X7R	Skeletal muscle cells	[13]
	UMSC-Exo/circHIPK3	NLRP3	Reduce miR421, upregulate FOXO3a, inhibit the downstream inflammatory pathway		[147]
	Sulforaphane	Nrf2, ARE	Activate Nrf2-ARE pathway and the antioxidant enzymes		[177]
	NAC	HIF-1α, NF-kB	Reduce HIF-1α transcription, caspase-1 and GSDMD activation		[152]
	BMP-7	HMGB1-TLR4	Attenuate hyperglycemia, inhibit inflammasomes priming		[182]
	TSA	HDAC1/2, P2X7R	Inhibit HDAC1/2 and reduce pyroptosis mediated by P2X7R		[153]
	Trimetazidine	PI3K/AKT	Upregulate PI3K/AKT pathway, inhibit activation of FoxO3a and NLRP3		[156]
	ECE and dieckol	RAGE, TLR4/AGE, HMGB1	Attenuate the combination of RAGE/TLR4 and their ligands	Gastrocnemius muscle cells	[155]
	MCC950	NLRP3	Decrease NLRP3, IL-1β, IL-18 level	Myocardial cells	[159]
	TRIM25 upregulation	NLRP1	Ubiquitinate NLRP1	Chondrocytes	[160]

### NLRP3 direct or indirect inhibitors

NLRP3 inflammasome is composed of an N-terminal pyrin domain, a central NACHT domain, and a C-terminal LRR domain. Hyperactivation of NLRP3 is the driving force of a wide range of pyroptosis-related diseases. Accordingly, several molecules inhibiting NLRP3 directly or indirectly have been reported.

The NACHT domain mediates NLRP3 oligomerization, ATP binding, and hydrolysis, which serves as essential processes in downstream reaction [15]. Interaction between the NACHT domain and drugs has been a potential treatment strategy. Sulfonylurea-containing compounds have been identified to block NLRP3 activation and IL-1β secretion. Glyburide, widely used in type 2 diabetes treatment, has been confirmed as the specific inhibition of NLRP3, not NLRP1 or NLRC4 [161]. Recent studies suggested that glyburide and bright blue G (BBG) downregulated non-canonical proteins, like caspase-4/-5/-11 and P2X7R, and attenuated IIM [13]. Interestingly, another hypoglycemic drug, metformin, has been shown to protect neurons and inhibit spinal cord injury by activation of the AMPK pathway and suppressing NLRP3 inflammasome, thus inhibiting pyroptosis [162]. MCC950 contains a diarylsulfonylurea domain, and this compound inhibits NLRP3 activation in both canonical and non-canonical pathways [163, 164]. MCC950 interacts with the NACHT domain directly and inhibits its ATPase activity. Similar to glyburide, this inactivation function is specific [164]. Therefore, MCC950 is widely used as a pyroptosis inhibitor. Both in vitro and in vivo experiments confirmed that MCC950 suppressed NLRP3, ASC, IL-18/-1β, and GSDMD levels, inhibited inflammatory reaction and Dox-induced myocardial injury [159], suggesting the treatment potential of MCC950 in muscle injury.

CY-09 is another inhibitor of NLRP3, which functions directly and selectively. In both surgical mice OA models and in vitro cultured chondrocytes, CY-09 attenuated TNF-α induced inflammatory stress and showed therapeutic effects in cartilage degeneration [165]. Wedelolactone phosphorylated Ser/Thr in NLRP3 and inhibited pyroptosis through potentiating PKA signaling on the MSU-induced RA model [53]. Interestingly, in addition to pharmacological NLRP3 blockers, Li et al. reported that moderate-intensity exercise-induced NLRP3 degradation by promoting the moderate activation of P2X7R, which in turn enhanced autophagy and inhibited pyroptosis [67]. This provides a strategy for nonpharmacologic therapy against the NLRP3 inflammasome.

### Hormone therapy

Based on the anti-pyroptotic capacity of some hormones, hormone therapy is considered a viable strategy for modulating inflammasome activation. Melatonin and its metabolites are broad-spectrum antioxidants and free radical scavengers. In the ox-LDL-induced atherosclerosis model, melatonin suppressed lncRNA MEG3, thus upregulating miR223 to inhibit NLRP3 formation [166]. Chen et al. reported that melatonin inhibited NF-kB and mitochondrial ROS production, thereby disrupting IL-1β/NF-kB/NLRP3 positive feedback loop and playing a protective role on the cartilage in IDD [141]. Another research further showed the regulatory effects of nicotinamide phosphoribosyl transferase on NLRP3 activation and matrix degradation in NP cells could be inhibited by melatonin [144]. For OA chondrocytes, melatonin inhibited oxidative stress by regulating mitochondrial and endoplasmic reticulum function to exert an impact on pyroptosis and alleviate the erosion of articular cartilage [74]. Similarly, A more recent study also demonstrated that melatonin attenuated mitochondrial oxidative stress, thereby restoring mitochondrial function and protecting ECM homeostasis in OA chondrocytes [167]. In addition, in human OA FLSs, estradiol exerted a protective effect by diminishing activated NLRP3 and IL-1β/-18 [83]. Besides, insulin may attenuate OP induced by diabetes mellitus. A study by Yan et al. found that in high glucose-induced osteoclasts, ROS, MAPKs-related protein, NF-kB, and NLRP3 were all upregulated, while insulin could reverse this effect [117].

### Exosomes treatment

Exosomes are vesicles released into the ECM containing bioactive cargoes involved in cell–cell communication [168]. Xu et al. isolated exosomes from bone marrow mesenchymal stem cells (BMSCs) and incubated them with OA chondrocytes. The results showed that BMSC-Exos repressed pyroptosis of chondrocytes by delivering miR-326 to target HDAC3 and enhancing the STAT1/NF-kB pathway [168, 169]. Another ncRNA, miR-26a-5p, was found to be delivered by human umbilical cord mesenchymal stem cells-derived exosomes (UMSC-Exo) and could degrade methyltransferase (METTL) 14 directly to protect NP cells from pyroptosis in IDD [170]. To cope with the rapid clearance and disruption of exosomes in IDD therapy, Xing et al. organized a thermosensitive acellular ECM hydrogel coupled with adipose-derived mesenchymal stem cell (ADSC) exosomes (dECM@exo) to release ADSC-derived exosomes persistently and inhibit NP cells from pyroptosis [171]. For DIMT, Dessouki et al. and Dargani et al. reported the protective effect of exosomes derived from embryonic stem cells (ES-Exos), such as inhibition of TLR4-NLRP3 pyroptosis pathway, increased synthesis of anti-inflammatory cytokines, and promotion of macrophage differentiation into M2 phenotype [157, 158]. For the skeletal muscle ischemic injury model, human UMSC-Exo released circHIPK3 that targeted on miR-421/FOXO3a pathway, resulting in the inhibition of pyroptosis and attenuated injury [147]. Similarly, EVs containing antioxidant proteins have been alternatives to protect against pyroptosis of NP cells in IDD. To strengthen the uptake process, Liao et al. constructed caveolae-associated protein 2 (Cavin-2) modified EVs and improved the endocytosis process [145]. Above all, the utilization of exosomes and EVs are limited to the clearance in ECM and endocytosis efficiency, which determines whether it can function consistently.

### Plant extracts

A variety of compounds extracted from plants exert biological effects on regulating NLRP3 and thus show therapeutic potential in MSDs. Licochalcone A (Lico A) could inhibit NLRP3 inflammasome via Nrf2/ HO-1/ NF-κB axis in LPS and surgery-induced OA mouse models [172]. Icariin (ICA) exerts a similar function by inhibiting NLRP3-caspase 1 axis [173]. Quercetin blocks oxidative stress-induced pyroptosis in the LPS-induced OA model [174], and morroniside exerts similar properties [49]. Another study investigated the anti-inflammatory effect of quercetin on macrophages, showing that quercetin suppressed TLR2/Myd88 and AMPK to downregulate macrophage pyroptosis [175]. In RA, Ge et al. established a collagen-induced arthritis model and found that punicalagin (PUN) retarded M1 polarization and pyroptosis through the NF-kB pathway [176]. Besides, paeoniflorin (MDP) is the main active ingredient of peony, and monomeric derivatives of MDP regulate ROS/GRK2/HIF-1α/NLRP3 pathway by inhibiting GRK2 phosphorylation in hypoxia-induced RA [101]. The effect of irisin has been explored in OP, and the regulation of Nrf2 and inhibition of NLRP3 reduced the incidence of postmenopausal OP [105]. Forskolin served as an adenylate cyclase activator and regulated the P2Y14R/cAMP/NLRP3 axis in MSU-induced pyroptosis of macrophages in Tohoku Hospital Pediatrics-1 (THP-1) cell line, which provides a novel insight to gouty arthritis treatment [123]. Last, natural antioxidants are involved in regulating muscle cell pyroptosis. Sun et al. reported that sulforaphane, as an activator of the Nrf-anti-oxidative response element (ARE) pathway, reduced NLRP3 activation, oxidative stress, and inflammatory response, thus participating in ischemia/reperfusion muscular injury [177].

### Molecules targeting other components of pyroptosis

Molecules targeting other components of pyroptosis pathways, including the upstream and downstream proteins, are potential treatments for MSDs. The combination of RAGE or TLR4 and HMGB1 or advanced glycation end products (AGE) activates the NF-kB pathway and leads to NLRP3 formation. Ecklonia cava extract (ECE) and dieckol attenuated Dexa-induced muscle atrophy by inhibiting this pathway [155]. GSDMD antagonists have shown their effectiveness in pyroptosis inhibition. Hu et al. identified disulfiram as a pore formation inhibitor by modifying Cys191/Cys192 in GSDMD. Although the processing of IL-1β and GSDMD still exists, the leakage of IL-1β and cellular contents leakage is blocked [178]. In 2021, Li et al. applied GSDMD antagonists (disulfiram) and HMGB1 antagonists (glycyrrhizic acid) on LPS/ATP-induced OA chondrocytes and revealed that these agents relieved inflammation and promoted proliferation, respectively. Another study based on the potassium oxonate-and monosodium urate-induced GA model also showed the anti-pyroptotic and anti-damage effects of disulfiram [124]. However, co-treatment of both had no such effect but led to oxidative stress [87]. In cigarette smoke- or disuse-induced muscle atrophy, HDAC1/2 protein upregulated P2RX7 and NLRP3, and HDAC inhibitor trichostatin A (TSA) reversed this effect [153]. Besides, TRIM25 ubiquitinates NLRP1 inflammasome, thus, overexpression of TRIM25 may exert a protective effect in Dox-induced cardiotoxicity [160]. Mitochondrial dysfunction of CD4+ T cells has a pro-inflammatory effect and promotes pyroptosis in RA, in which mtDNA leakage and caspase-1 activation may play a vital role. Wang et al. constructed a chimeric NSG mice model and found that overexpression of DNA repair nuclease-MRE11A stabilized mtDNA structure and maintained homeostasis, which showed a similar effect to the application of caspase-1 inhibitor VX-765 [93]. Generally, RA is characterized by a decrease in the PH of the joint fluid and increased H+ level could open ASICs to affect Na+ and Ca2+ permeability. Wu et al. reported that ASIC inhibitors, amiloride and psalmotoxin-1 (PcTx-1), impaired Ca2+ influx, and calcium chelating agent-BAPTA-AM reduced cytosolic calcium concentration, through which both agents showed joint protection in RA [97]. Moreover, previous studies showed that Ca2+ level interacted with the pyroptosis process [179], thus, it can be speculated that ASIC may mediate pyroptosis through the upregulation of Ca2+. Hypoxia-induced ROS leads to muscle dysfunction, and antioxidants like N-acetylcysteine (NAC) or sulforaphane (SFN) have positive therapeutic effects by targeting suppression of HIF-1α synthesis or upregulation of the Nrf2- ARE pathway [152, 180]. The inhibitory effect of Dexa on the phosphorylation of PI3K/AKT/FoxO3a pathway is proven to be a reason for the up-regulation of NLRP3-GSDMD pyroptosis pathway and muscle atrophy. Wang et al. reported that trimetazidine reversed this inhibitory effect, thereby downregulating pyroptosis and alleviating Dexa-induced muscle atrophy [156].

### Regulatory of endogenous cytokines

Regulation and application of endogenous cytokines may provide a novel intervention strategy. Bone morphogenetic proteins (BMPs), as a subdivision of TGF-β, are signal molecules that affect many physiological processes [180]. Previous studies confirmed that BMP-2 mediated osteogenic differentiation of MSCs induced by simvastatin in OP [181]. Similarly, in streptozotocin-induced diabetic myopathy, BMP-7 retarded pyroptosis of muscle cells [182]. Collectively, these findings suggest that BMPs, as pyroptosis inhibitors, may be a potential alternative for MSD treatment. A homeostatic CXC chemokine, stromal cell-derived factor-1 (SDF-1), has been explored in OA pathogenesis and treatment. OA synoviocytes expressed significantly lower SDF-1 and higher pyroptosis biomarkers. SDF-1 activated the AMPK/PI3K-mTOR signaling pathway and suppressed NLRP3, suggesting that SDF-1 serves as a potential agent for OA treatment [183].

## Future perspectives and conclusions

Previous studies have revealed multiple mechanisms of pyroptosis and discussed the possible intervention strategies. However, it should be noted that some studies may be contradictory to the results mentioned above. For instance, Busso et al. demonstrated that IL-α and IL-1β are not key mediators in murine OA models [184]. Another study by Bougault and colleagues also reported that knockout of NLRP3 and inhibition of caspase-1 or IL-1β did not inhibit the expression of MMP-3/-9/-13, indicating a negative role in ECM degradation and MSDs development [185]. Therefore, further validation of these studies is still demanded. In addition, there are some prospects for future research directions.

First, various cell death pathways interplay with each other to participate in the occurrence and development of MSDs. Treatment strategies should focus on co-effectors of different cell death pathways, rather than only lay interventions on a single pathway. One critical target among the crosstalk of cell deaths is caspase-8, which could induce apoptosis and pyroptosis, and inhibit necroptosis [38]. Other caspases shared in different cell death pathways need to be explored for their unique roles. Channels on cell membranes formed by GSDMD, MLKL, or Panx-1 induce K+ efflux and thus activate NLRP3 inflammasome [41, 48], while autophagy mostly shows an inhibitory effect on pyroptosis [52]. Thus, the effect of pyroptosis on MSDs should be viewed as the systemic regulation of multiple pathways in order to offer better treatment with enhanced efficacies and minimal adverse effects.

Second, exosomes have provided a novel therapeutic strategy for MSDs. However, its clinical utilization faces a number of challenges. First of all, its stability in ECM and the ability to bind with target cells could largely affect its efficacy. Currently, several methods have been attempted to solve these problems, either by constructing a matrix that preserves exosomes or by modifying vesicle membranes [145, 171]. Besides, other active components in exosomes, such as miRNA and antioxidants, need to be further explored. For example, the regulatory effects of miRNAs that inhibit NLRP3 on other physiological processes need to be studied to clarify the possible side effects.

Third, most of the current studies are based on in vitro or in vivo experiments, while clinical trials are still insufficient. On the one hand, genes involved in pyroptosis between mice and humans are different. For instance, caspase-11 in the non-canonical pathway only exists in rodents, but not in humans. Accordingly, cellular or animal models may not mimic the internal microenvironment in humans, which may introduce potential bias to the clinical interpretation of these studies. On the other hand, many treatments are not yet in clinical use. This requires the development of clinical drugs and more extensive research based on basic and pre-clinical investigations.

In this review, we have concluded the canonical, non-canonical, and alternative pathways in pyroptosis, and discussed the crosstalk between pyroptosis and other regulated cell death pathways, like apoptosis, necroptosis, and autophagy. Then we summarized the pathological role of pyroptosis in several MSDs, including OA, RA, OP, GA, AS, IDD, IIMs, and muscle injury. The current therapeutic strategies that target pyroptosis in these MSDs were depicted, most of them are based on direct or indirect inhibition of NLRP3 by exosomes, hormones, and plant extracts. It is speculated that research on the correlation between pyroptosis and MSDs will flourish continuously with an improving understanding of its underlying mechanisms and advancing practical application, which will offer more clinical strategies for diagnosis and treatment in the near future.

## Data Availability

The data used to support this study are included in the article.
